# Characterization and comparative analysis among plastome sequences of eight endemic *Rubus* (Rosaceae) species in Taiwan

**DOI:** 10.1038/s41598-020-80143-1

**Published:** 2021-01-13

**Authors:** JiYoung Yang, Yu-Chung Chiang, Tsai-Wen Hsu, Seon-Hee Kim, Jae-Hong Pak, Seung-Chul Kim

**Affiliations:** 1 Department of Biology, School of Life Sciences, BK21 FOUR KNU Creative BioResearch Group, Kyungpook National University, Daegu, 41566 Republic of Korea; 2 Department of Biological Sciences, National Sun Yat-Sen University, Kaohsiung, 80424 Taiwan; 3 Taiwan Endemic Species Research Institute, 1 Mingshen East Road, Chichi Township, Nantou, 55244 Taiwan; 4 Department of Biological Sciences, Sungkyunkwan University, 2066 Seobu-ro, Suwon, Gyeonggi-do 16419 Republic of Korea

**Keywords:** Plant sciences, Plant evolution, Taxonomy, Molecular evolution

## Abstract

Genus *Rubus* represents the second largest genus of the family Rosaceae in Taiwan, with 41 currently recognized species across three subgenera (*Chamaebatus*, *Idaoeobatus*, and *Malochobatus*). Despite previous morphological and cytological studies, little is known regarding the overall phylogenetic relationships among the *Rubus* species in Taiwan, and their relationships to congeneric species in continental China. We characterized eight complete plastomes of Taiwan endemic *Rubus* species: subg. *Idaeobatus* (*R*. *glandulosopunctatus*, *R*. *incanus*, *R*. *parviaraliifolius*, *R rubroangustifolius*, *R*. *taitoensis,* and *R*. *taiwanicolus*) and subg. *Malachobatus* (*R*. *kawakamii* and *R*. *laciniastostipulatus*) to determine their phylogenetic relationships. The plastomes were highly conserved and the size of the complete plastome sequences ranged from 155,566 to 156,236 bp. The overall GC content ranged from 37.0 to 37.3%. The frequency of codon usage showed similar patterns among species, and 29 of the 73 common protein-coding genes were positively selected. The comparative phylogenomic analysis identified four highly variable intergenic regions (*rps16*/*trnQ*, *petA*/*psbJ*, *rpl32*/*trnL*-UAG, and *trnT*-UGU/*trnL*-UAA). Phylogenetic analysis of 31 representative complete plastomes within the family Rosaceae revealed three major lineages within *Rubus* in Taiwan. However, overall phylogenetic relationships among endemic species require broader taxon sampling to gain new insights into infrageneric relationships and their plastome evolution.

## Introduction

Taiwan originates from the continental Taiwan-Ryukyu Archipelago, lying on the western rim of the Pacific Ocean approximately 150 km from the southeastern coast of China, separated by the Taiwan Strait. Taiwan was repeatedly connected and disconnected during the glacial and interglacial periods of the Pleistocene glaciation cycles, which provided opportunities for plant colonization and isolation between the islands and the mainland^1,2^. In addition, Taiwan provided refugia for northern species that migrated south during the glacial periods. These striking biogeographic events and topographic heterogeneity in the island’s environment resulted in floristic affinity between Taiwan and mainland China, as well as the formation of unique floristic elements in Taiwan. For example, approximately 52% of the ca. 4,000 native vascular plant species are closely related to the mainland China, while ca. 26% of natives (1,067 taxa) are endemic to Taiwan^3^. A combination of multiple colonization events from geographically close source areas, followed by subsequent speciation on the island without splitting events (i.e., phyletic speciation), and in situ adaptive and nonadaptive speciation after colonization on island likely explains the high endemism of flora in Taiwan^2^. In particular, several distinctive vegetation zones provided opportunities for the diversification of several rich groups of endemic species, ranging from tropical and coastal evergreen forests, to subalpine shrubs and alpine tundra^4–6^.

The genus *Rubus* L., with ca. 700 species, is distributed worldwide and is abundant in the Northern Hemisphere, with very few species occurring in the Southern Hemisphere^7^. Focke established the widely adopted *Rubus* infrageneric classification system that recognizes 12 subgenera^8–10^, and several attempts have been made to unravel the overall phylogeny within the genus as well as the role of hybridization events^11–16^. Approximately 41 species from three subgenera are currently recognized in Taiwan; i.e., *Chamaebatus* (3 species), *Idaoeobatus* (27 species), and *Malochobatus* (11 species). Among these species, approximately 40% (15 species) are considered endemic to Taiwan, while the remaining species occur in mainland China (23 species; 57.5%), Japan (12 species; 30%), and the Philippines (6 species; 15%)^17^. Despite previous floristic and cytological studies, as well as reports of new natural hybrid taxa^6,17,18^, very little is known regarding the evolutionary history among *Rubus* species in Taiwan and their phylogenetic relationships to congeneric species in continental China and adjacent countries.

Chloroplasts are essential organelles in plant cells that serve as metabolic centers in green plants and encode many key proteins that are involved in photosynthesis and other metabolite syntheses. Phylogenetic relationships of major plant groups at various taxonomic levels based on plastid genomes have been greatly elucidated by sequence polymorphisms or by hypervariable microsatellites (simple sequence repeats) as efficient genetic markers^19–21^. In addition, several chloroplast gene loci (e.g., *matK* and *rbcL*) have proven useful as DNA barcode markers for species discrimination that provide valuable information to develop conservation strategies and biodiversity assessments^22,23^. Achieving adequate resolutions on the basis of the phylogenetic analyses of several concatenated regions has been difficult, especially for recently diverged plant species, because of limited sequence variations in several coding and noncoding regions of chloroplast DNA^24–26^. With the advent of next-generation sequencing (NGS) tools, considerable genome-wide variations in phylogenomics have significantly enhanced our understanding of patterns and processes in plant evolution, especially at lower taxonomic levels^27–32^. Since the first report of three partial *Rubus* plastomes being part of the Rosaceae phylogeny^33^, several complete chloroplast genomes belonging to different subgenera, i.e., *Anoplobatus* and *Idaeobatus*, have been recently reported, and useful hotspot regions for phylogenetic analysis have been suggested^34–39^. Our knowledge of plastome structure and evolution was primarily synthesized from the subgenus *Idaeobatus* from the infrageneric classification system of *Rubus* by Focke^8–10^, with the exception of two species from subg. *Anoplobatus* and one species from subg. *Cylactis*, based on species from Korea and China. Thus, we know very little regarding the plastome structure and evolution of other subgenera and also from other geographical regions in East Asia.

Therefore, we aimed to determine the complete plastomes of eight *Rubus* species endemic to Taiwan, including six species from subgenus *Idaeobatus* and two species from subgenus *Malacobatus*. Although few other endemic species of *Rubus* belong to subgenus *Chamaebatus*, we were not able to include any representatives in current study. The comparative analysis of these eight plastomes, alongside the other previously reported plastomes within the genus will allow us to elucidate the genome structure, gene order, and gene contents, eventually providing an opportunity to study plastid evolution across groups. In addition, this study will shed light on the plastome structure and evolution of insular endemic species of the Taiwan-Ryukyu Archipelago. Lastly, the results from this study will aid in the development of useful chloroplast markers from hotspot regions, facilitating increased resolution of phylogenetic relationships among closely related species of *Rubus*.

## Results

### Genome size and features

**Figure 1:**
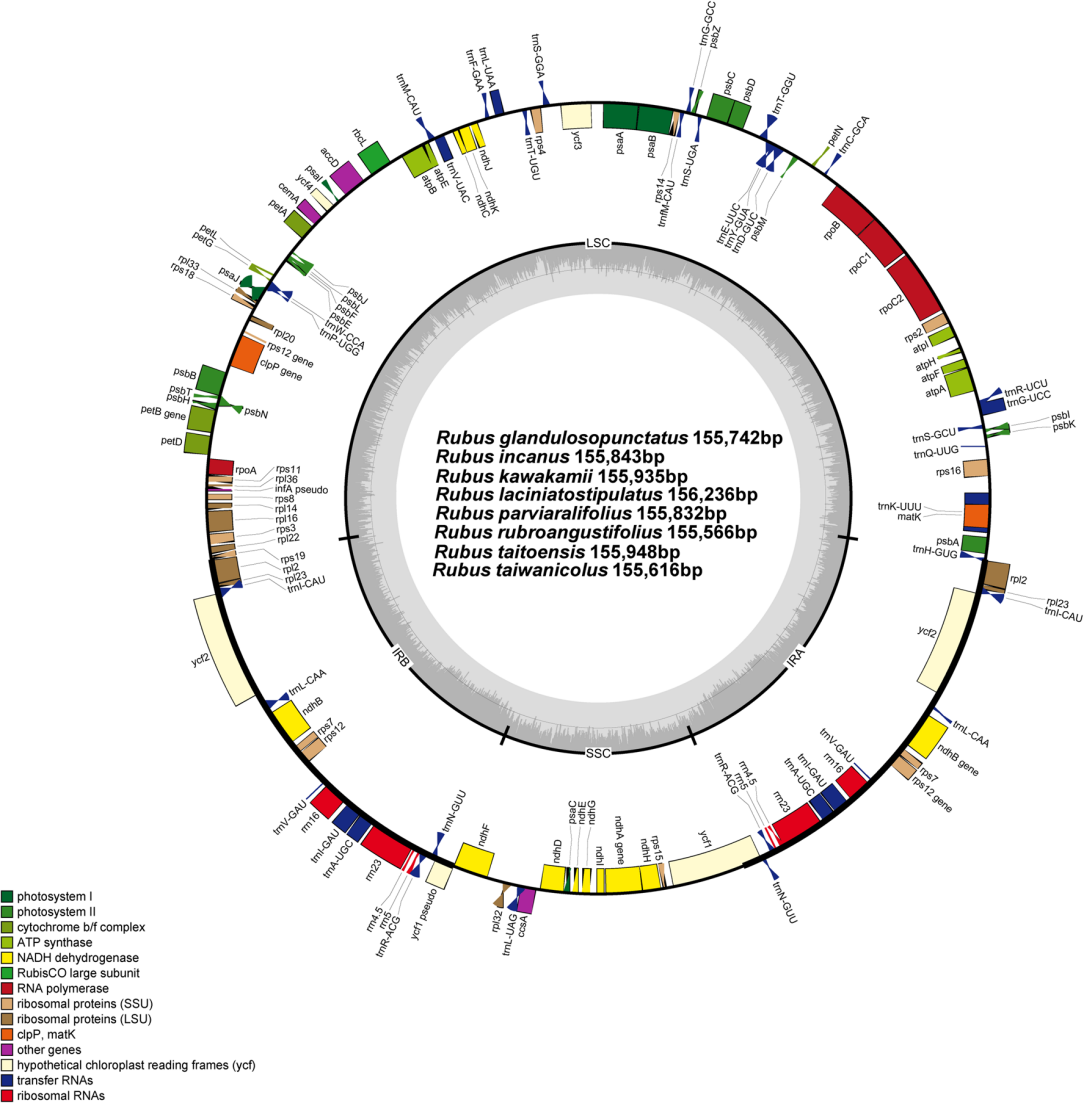
The eigth endemic *Rubus* plastomes in Taiwan. The genes located outside of the circle are transcribed clockwise, while those located inside are transcribed counterclockwise. The gray bar area in the inner circle denotes the guanine–cytosine (GC) content of the genome, whereas the lighter gray area indicates the adenosine–thymine (AT) content of the genome. Large single copy, small single copy, and inverted repeat are indicated with LSC, SSC, and IR, respectively. Gene map was generated with the OrganellarGenomeDRAW (OGDRAW) 1.3.1. (https://chlorobox.mpimp-golm.mpg.de/OGDraw.html.).

For the first time, the complete plastomes of eight endemic *Rubus* species from Taiwan were characterized: six species from subg. *Idaeobatus* and two species from subg. *Malachobatus*. The size of complete plastome sequences ranged from 155,566 bp (*R*. *rubroangustifolius*) to 156,236 bp (*R*. *laciniatostipulatus*). The plastomes were highly conserved with no structural variations or content rearrangements despite their representations from different subgenera (Fig. 1). The eight plastomes of Taiwanese endemic *Rubus* contained 131 genes: 84 protein-coding, eight ribosomal RNA, and 37 transfer RNA genes. Compared with previously reported GC content of *Rubus* plastomes (37.1%)^35–39^, the overall GC content ranged from 37.0% (*R*. *glandulosopunctatus, R. rubroangustifolius,* and *R. taiwanicolus*) to 37.3% (*R. incanus, R. parviaralifolius,* and *R*. *taitoensis*), summarized in Table 1. A total of 17 genes were duplicated in the inverted repeat regions, including seven tRNA, four rRNA, and six protein-coding genes. Fifteen genes (*ndhA, ndhB, petB, petD, rpl2, rpl16, rpoC1, rps12, rps16, trnA-*UGC*, trnG-*UCC*, trnI-*GAU*, trnK-*UUU*, trnL-*UAA*,* and *trnV-*UAC) contained one intron, whereas *clpP* and *ycf3* each contained two introns.

**Table 1:** Summary of the characteristics of the eight endemic *Rubus* chloroplast genomes in Taiwan.

Taxa	*R. glandulosopunctatus*	*R. incanus*	*R. kawakamii*	*R. laciniatostipulatus*	*R. parviaralifolius*	*R. rubroangustifolius*	*R. taitoensis*	*R. taiwanicolus*
Total cpDNA size (bp)	155,742	155,843	155,935	156,236	155,823	155,566	155,948	155,616
GC content (%)	37.0	37.3	37.2	37.2	37.3	37.0	37.3	37.0
LSC size (bp)	85,526	85,071	85,548	85,862	85,058	85,371	85,175	85,408
IR size (bp)	25,749	25,992	25,772	25,762	25,992	25,749	25,992	25,742
SSC size (bp)	18,718	18,788	18,843	18,850	18,790	18,697	18,789	18,724
Number of genes	131	131	131	131	131	131	131	131
Number of protein-coding genes	84	84	84	84	84	84	84	84
Number of tRNA genes	37	37	37	37	37	37	37	37
Number of rRNA genes	8	8	8	8	8	8	8	8
Number of duplicated genes	17	17	17	17	17	17	17	17
Accession number	MT274118	MT274119	MT274120	MT274121	MT274122	MT274123	MT274124	MT274125

*LSC* large single copy region, *IR* inverted repeat, *SSC* small single copy region.

**Figure 2:**
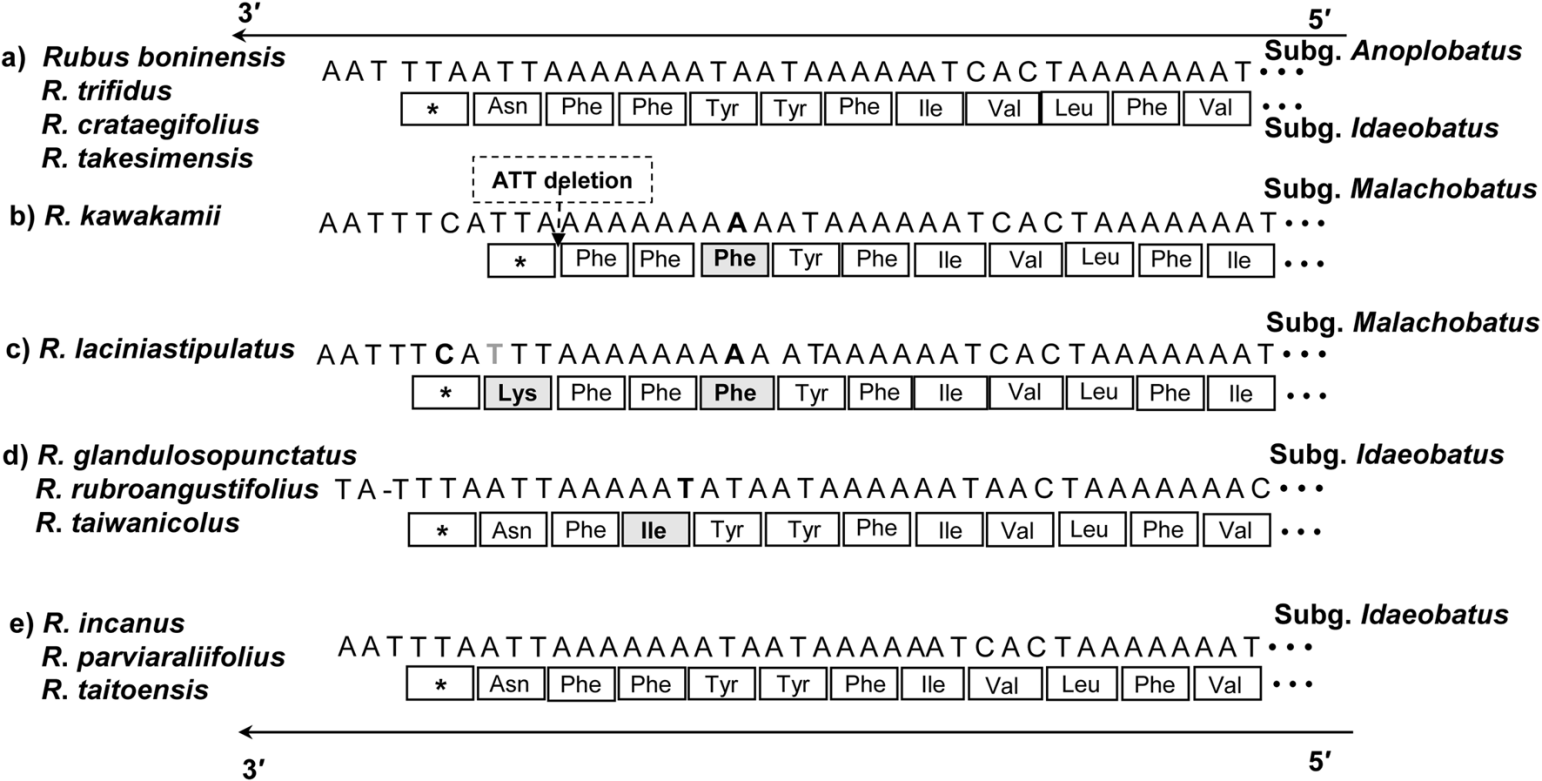
The 3′ region of *ndhF* genes of 12 *Rubus* species. In each lane, aligned DNA sequence data are shown on the top, box present the amino acid sequences that are coded from the DNA sequences, and asterisk (*) denotes the terminal codons. The mutated residues and amino acids are bolded.

A partial *ycf1* gene (1,107–1,248 bp) was located in the IRb/SSC junction region, while the complete *ycf1* gene (5,820–5,862 bp) was located in the IR region at the SSC/IRa junction. The *infA* gene of the eight Taiwanese endemic *Rubus* plastomes located in the LSC region became a pseudogene. Interestingly, the highly conserved group II intron of *atpF* was lost, and a frameshift mutation via ATT deletion of the *ndhF* gene was identified in *R*. *kawakmii* of subg. *Malachobatus* (with a CDS size variation length of 2,241 bp), and caused early termination of translation (Fig. 2b). Also, it displayed point mutations, altering from transcriptions of tyrosine (Tyr) to phenylalanine (Phe). Two other point mutations on the *ndhF* gene altered phenylalanine (Phe) to isoleucine (Ile) and tyrosine (Tyr) to phenylalanine (Phe); the former mutation was in *R. glandulosopunctatus, R. rubroangustifolius, R. taiwanicolus* (Fig. 2d) and the latter in *R. laciniastipulatus* (Fig. 2c). Additionally, a point mutation was detected in *R. laciniastipulatus*, altering asparagine (Asn) to lysine (Lys) (Fig. 2c). Three Taiwanese *Rubus* plastomes from subg. *Idaeobatus* (*R. incanus, R. parviaraliifolius,* and *R. taitoensis*) contained the same consistent distribution of amino acids, sequences (Fig. 2e), and the CDS length of 2242 bp as four *Rubus* plastomes of two subgera *Idaeobatus* and *Anoplobatus* sampled from the Korean peninsula and Japan (Fig. 2a)^39^.

**Figure 3:**
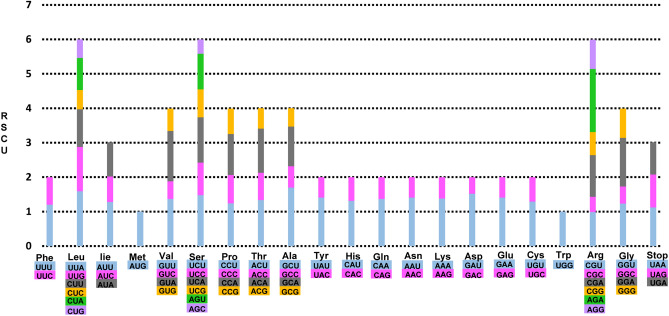
Codon distribution and relative synonymous codon usage (RSCU) in eight endemic *Rubus* in Taiwan. The RSCU values are represented on the y-axis, while the codon families for each amino acid are denoted on the x-axis.

The frequency of codon usage of the eight complete Taiwanese endemic *Rubus* plastomes was calculated for the cp genome on the basis of the sequences of protein-coding genes and tRNA genes (Fig. 3). The average number of codon usage ranged from 24,134 (*R. kawakamii*; subg*. Malachobatus*) up to 26,093 (*R*. *parviaralifolius*; subg*. Idaeobatus*), but the distribution of codon type was consistent. Excluding the AUC codon usage of *trn*V-GAU in *R*. *laciniatostipulatus* (subg. *Malachonatus),* similar patterns of cp genes and codon usage were detected amongst the eight endemic *Rubus* species (Supplementary Table 1). The codon usage in eight Taiwanese endemic *Rubus* plastomes was biased toward high RSCU values of U and A at the third codon usage. A similar phenomenon was observed in other angiosperms^40^ and algal lineages^41^.

The RNA editing prediction in eight Taiwanese *Rubus* endemics indicated 69 sites in total with the same cut-off value, and 19 out of the 35 protein coding-genes (Supplementary Table 2). Those genes included photosynthesis genes (*ndA, ndhB, ndhD, ndhF, ndhG, petB, psaI, psbE,* and *psbF*), self-replication genes (*rpl23, rpoA, rpoB, rpoC2, rps2, rps14,* and *rps16*) and others (*accD*, *clpP*, and *matK*). No RNA editing sites at *ndhF* and *ndhG* genes were found in *R*. *glandulosopunctatus*, *R*. *rubroangustifolius*, and *R. taiwanicolus*. In addition, RNA editing sites at the *rpoC1* gene was observed in *R*. *kawakamii*. Compared with other species, the *ndhA* gene in *R*. *glandulosopunctatus* showed exceptionally higher frequencies (i.e., five times) in RNA editing sites. The *ndhB* gene had the highest number of potential editing sites (a total of 11 sites), followed by the *ndhD* gene (a total of eight sites). It showed consistent results from previous reports^42–44^, except for *R*. *glandulosopunctatus* that had the second highest number of potential editing sites in *ndhA* (10). All editing sites showed base transition from cytosine (C) to thymine (T), and the most frequent transition serine (Ser) conversion to leucine (Leu) (Fig. 3). Consequently, the amino acids with hydrophobic chains (isoleucine, leucine, methionine, and phenylalanine) formed in 88.6% of the 29 RNA editing sites.

### Comparative analysis of genome structure

**Figure 4:**
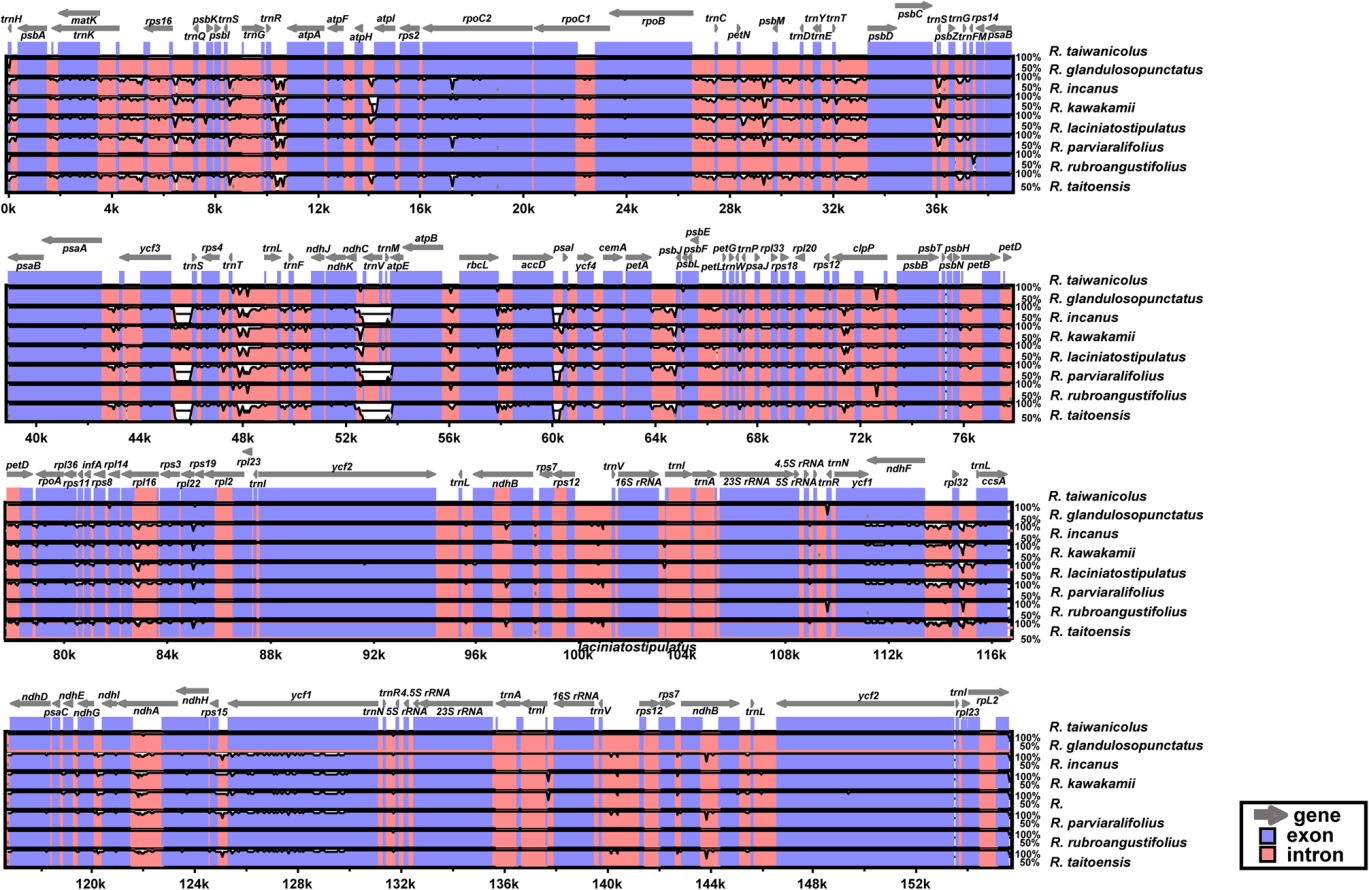
Visualization of alignment of eight *Rubus* species chloroplast genome sequences. The VISTA-based identity plots using mVISTA in Shuffle-LAGAN (LAGAN toolkit version 2.0; http://lagan.stanford.edu/glocal) show the sequence identity of eight Taiwan endemic *Rubus* species. Vertical scale indicates the percent identity from 50 to 100%. Coding and non-coding regions are in blue and pink, respectively. Gray arrows above the alignment indicate the position and direction of each gene.

The eight complete plastome sequences of endemic *Rubus* species in Taiwan were plotted using mVISTA analysis using the annotated *R*. *taiwanicolus* plastome as a reference (Fig. 4). The results indicated that the LSC region was most divergent, that the two IR regions were highly conserved, and that the non-coding regions were more divergent and variable than the coding regions. In addition, the *R*. *incanus, R. parviaraliifolius* and *R. taitoensis* plastomes showed high sequence similarity (i.e., 97% sequence identity; 149,200 bp identical sites) to the *R*. *taiwanicolus* plastome, while *R*. *kawakamii* was least similar (97% sequence identity; 149,178 bp identical sites) to *R*. *taiwanicolus*.

**Figure 5:**
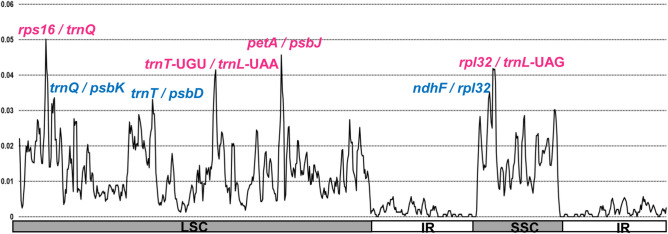
Sliding window analysis of the eight whole chloroplast genomes of *Rubus* species in Taiwan. X-axis: position of the window midpoint, Y-axis: nucleotide diversity within each window.

The sliding window analysis using DnaSP program revealed highly variable regions in the continental Island endemic taxa of the *Rubus* chloroplast genome (Fig. 5). As the eight plastomes of *Rubus* from Taiwan were compared, the average value of nucleotide diversity (*Pi*) over the entire cp genome was 0.010. The most variable region was the *rps16*/*trnQ* intergenic region with a 0.05018 *Pi* value. Also, highly variable regions included three other intergenic regions: *petA*/*psbJ* (*Pi* = 0.04567), *rpl32*/*trnL*-UAG (*Pi* = 0.04165), and *trnT*-UGU/*trnL*-UAA (*Pi* = 0.04147). Therefore, four highly variable regions with a *Pi* value greater than 0.04 identified in eight endemic *Rubus* plastomes in Taiwan can be useful for population genetic and phylogeographic study.

**Figure 6:**
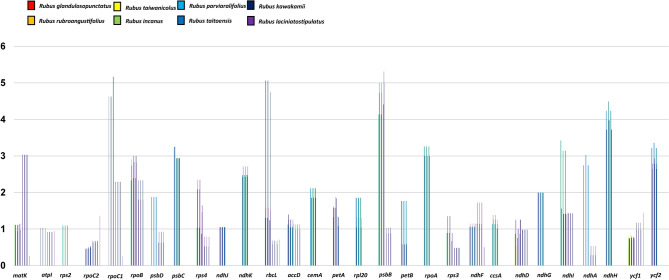
The Ka/Ks ratio of 73 protein-coding genes of cp genomes from eight endemic whole chloroplast genomes of *Rubus* species in Taiwan. X-axis: Ka/Ks ratio, Y-axis: 29 protein-coding genes with the Ka/Ks ratio of > 1.

The positive selection analysis using DnaSP program with synonymous and nonsynonymous substitution options revealed positively selected genes (Fig. 6). Overall, the average Ka/Ks ratio of the 73 common protein-coding genes in the eight endemic plastomes was 0.45. For each conserved gene, a total of 44 (out of 73 genes) had an average Ka/Ks ratio below 1 for the eight comparison groups, suggesting that these genes were subjected to strong purifying selection pressures in the *Rubus* chloroplast. The Ka/Ks ratio of > 1 had a total of 29 genes out of 73, suggesting that these genes were positively selected in the eight endemic plastomes in Taiwan. Those genes included one ATP subunit gene (*atpI*), three photosystem subunit genes (*psbB, psbC,* and *psbD*), two cytochrome b6/f complex gene (*petA* and *petB*), eight NADH oxidoreductase genes (*ndhA*, *ndhD*, *ndhF*, *ndhG*, *ndhH*, *ndhI*, *ndhJ,* and *ndhK*), Rubisco gene (*rbcL*), four encoded DNA dependent RNA polymerase genes (*rpoA, rpoB, rpoC1,* and *rpoC2*), four ribosomal subunit genes (*rpl20*, *rps2*, *rps3*, and *rps4*), maturase gene (*matK*), one subunit Acetyl-CoA carboxylase gene (*accD*), one c-type cytochrome synthesis gene (*ccsA*), one envelop membrane protein gene (*cemA*), and two unknown genes (*ycf1* and *ycf 2*). Surprisingly, our results from the Taiwanese endemic *Rubus* suggested that ca. 40% of the protein-coding genes underwent positive selection pressures.

### Phylogenetic analysis

**Figure 7:**
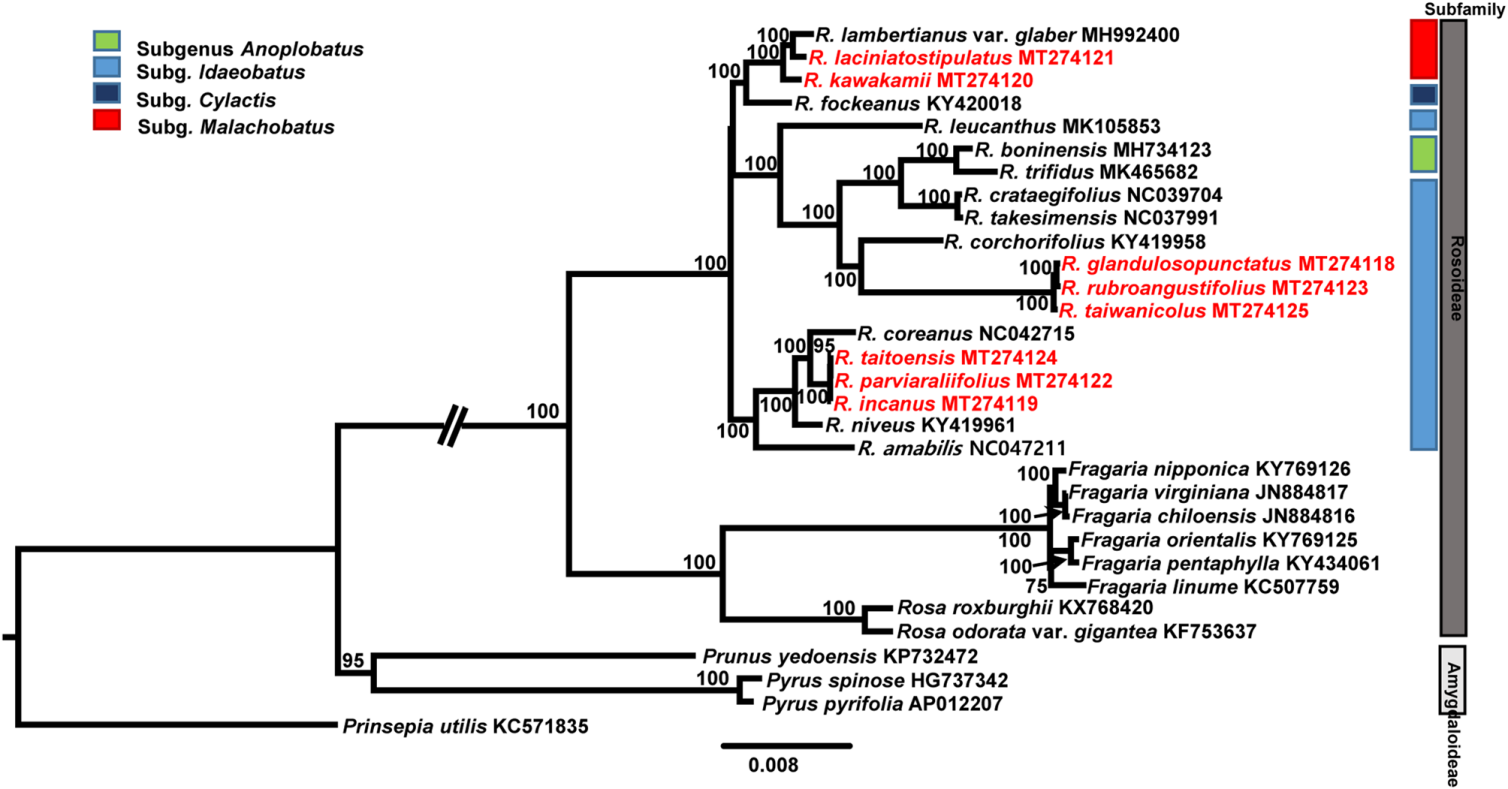
The maximum-likelihood (ML) tree using IQ-TREE v. 1. 4. 2. (http://www.iqtree.org/) inferred from 31 representative species of Rosaceae. The complete plastomes of eight *Rubus* endemic species from Taiwan are labeled in red. The bootstrap value based on 1,000 replicates is shown on each node. *Prinsepia utillis* was used as an outgroup and columns on the right indicate two subfamilies of Rosaceae, Rosoideae and Amygdaloideae, and subgeneric classification of *Rubus* (*Anoplobatus*, *Idaeobatus*, *Cylactis*, and *Malachobatus*).

Maximum likelihood (ML) analysis conducted on the best-fit model of “TVM + F + R4” revealed the first preliminary phylogenetic relationships among endemic species in Taiwan (Fig. 7). Phylogenetic analysis of 31 representative plastomes within the family Rosaceae supported both the monophyly of *Rubus* (100% bootstrap support) and the sister relationship between *Rubus* and the clade containing *Fragaria* and *Rosa* (100% bootstrap support). Based on limited available complete plastome sequences of *Rubus* from four subgenera, *Anoplobatus* and *Malachobatus* appear to be monophyletic, while *Idaeobatus* is not monophyletic (Fig. 7). Subgenus *Cylactis* (*R*. *fockeanus*) is sister to *Malachobatus* (*R*. *laciniatostiplatus*, *R*. *lambertianus* var. *glaber*, *R*. *kawakamii*; 100% bootstrap support), and six species of *Idaeobatus* (*R. amabilis*, *R. coreanus*, *R. niveus*, *R. taitoensis*, *R. parviaralifolius*, and *R. incanus*) are located basal lineage within genus *Rubus* clade. The continental progenitor-insular derivative species pair in Japan, *R. boninensis* and *R. trifidus*, are sister to the other progenitor-derivative species pair *R. crataegifolius* and *R. takesimensis* in Korea. Subgenus *Anoplobatus* appears to be deeply embedded within *Idaeobatus* (Fig. 7). Results from the phylogenetic relationships and positions of the eight Taiwanese endemics indicated that *R. glandulosopunctatus*, *R. rubroangustifolius*, and *R. taiwanicolus* formed a monophyletic group and were sister to *R. corchorifolius* (100% bootstrap support). The other clade of Taiwanese endemics including *R. taitoensis*, *R. parviaraliifolius*, and *R. incanus*, is sister to *R. coreanus*, while *R. laciniatostipulatus* and *R. kawakamii* in *subg. Malachobatus* is sister to *R. fockeanus* in subg. *Cylactis*. These suggest that endemic species of *Rubus* in Taiwan most likely have evolved at least three times from different lineages (Fig. 7), requiring further confirmation based on rigorous sampling from both continental and island species.

## Discussion

### Chloroplast genome structure and evolution in Taiwanese *Rubus* endemics

For the first time, we characterized the eight complete plastomes of Taiwan endemic *Rubus* species, including two species from subg. *Malachobatus*. The size of the complete plastome sequences are highly conserved with the total length ranging from 155,566 to 156,236 bp (Table 1). In addition, despite their representations from two subgenera (*Idaeobatus* and *Malachobatus*), the plastomes are highly conserved, with no structural variations or content rearrangements. Interestingly, the highly conserved group II intron of *atpF* gene was lost in all eight plastomes regardless of their subgeneric assignments, as we demonstrated previously in the case of *R. boninensis, R. crataegifolius, R. takesimensis* and *R. trifidus*^35,39^. Within the major lineages of the family Rosaceae^45^, we found the complete *atpF* gene in members of the newly circumscribed subfamily Amygdaloideae, such as *Prunus* (KP732472), *Pyrus* (HG737342, AP012207), and *Malus* (NC040170, NC031163). However, loss of the *atpF* intron was also detected in other members of Rosoideae, such as *Fragaria* (KY769126, 769125, 434061), *Hagenia* (KX0088604), *Potentilla* (HG931056), and *Rosa* (KY419918, KX768420, KY419934). It still remains to be determined whether the loss of the *atpF* intron, that frequently occurs in *Rosa* and all subgenera of *Rubus,* has occurred in other major lineages within the family Rosaceae.

In this study, we detected mutations in the 3′ region of the *ndhF* gene; which is known to have frameshift mutations and alterations on transcription termination, as a result of higher substitution rate, a wide range of insertion and deletion (indel) variations, a low homoplasy rate, and a high AT content^46,47^. In comparative phylogenomic analysis of genus *Rosa* sect. *Synstylae*, Jeon and Kim^30^ revealed frameshift and point mutations on the 3′ end of the *ndhF* gene. However, our previous study on the comparative analysis of four *Rubus* plastomes (two from each subgenus *Anoplabatus* and *Idaeobatus*) only showed nucleotide substitutions and transcription alterations without size variations^39^. A frameshift mutation via ATT deletion that caused early termination and translation was identified in *R*. *kawakamii* (subg. *Malachobatus*) from the eight Taiwan endemic *Rubus* plastomes (Fig. 2). Although the other endemic *R. laciniatostipulatus* belongs to the same subg. *Malachobatus*, it only showed a point mutation, which altered transcriptions from asparagine (Asn) to lysine (Lys) and tyrosine (Tyr) to phenylalanine (Phe). We also detected point mutations in other endemic *Rubus* in Taiwan for the same 2,242 bp sequences, which altered transcripts from tyrosine (Tyr) to phenylalanine (Phe), phenylalanine (Phe) to isoleucine (Ile), tyrosine (Tyr) to phenylalanine (Phe), and asparagine (Asp) to lysine (Lys). Furthermore, three Taiwanese endemic *Rubus* plastomes in subg. *Idaeobatus* (*R. incanus, R. parviaraliifolius,* and *R. taitoensis*) showed the same distribution of amino acids and sequence lengths (2,242 bp) with the four previously analyzed members of *Rubus* plastomes (two in subg. *Idaeobatus* and two in *Anoplobatus*)^39^. It appears that these changes are neither subgenus specific nor geographically confined to the East Sea (Ulleung Island), the northwestern Pacific Ocean (Bonin Islands), and the western rim of Pacific Ocean (Taiwan).

The codon usage bias, which could be manifested primarily by the balance between mutational bias and natural selective forces, provide crucial information to our understanding of molecular evolution and environmental adaptation^41,48,49^. In the case of *R. trifidus* (continental progenitor)—*R. boninensis* (insular derivative) species pairs in subg. *Anoplobatus*, we found similar patterns in codon usage with some exceptions^39^. When compared with the pair of *R. trifidus*-*R. boninensis*, AUG (*trnfM-*CAU*, trnI-*CAU*,* and *trnM-*CAU), UCA (*trnS-*UGA), UAG (no usage), and CAA codon usage (*trnQ-*UUG*)* showed different patterns in *R. crataegifolius*-*R. takesimensis* species pairs. When compared with these two species pairs, AUG (*trnfM-*CAU*, trnI-*CAU*,* and *trnM-*CAU), CAA (*trnQ-*UUG), and UCA (*trnS-*UGA) codon usage of the eight Taiwanese *Rubus* endemics showed similar patterns to *R. cratageifolius*, *R. takesimensis*, and *R. trifidus* from Korea. The specific codon usage amongst eight endemic *Rubus* was detected in *R*. *laciniatostipulatus* (subg. *Malachobatus*) with AUG codon usage (*trn*V*-*GAU). The UAG codon usage (stop codon) was similar only to *R. crataegifolius* and *R. takesimensis* on Ulleung Island, and *R. boninensis* on Bonin Islands. *Rubus trifidus* showed a different UAG codon usage of *trnI-*CAT. However, the Bonin Islands endemic *R. boninensis* showed different patterns of codon usage at AUG (*trnfM-*CAU and *trnI-*CAU), CAA (*trnK-*UUG) and UCA (no usage). Given that *R. boninensis* occurs on Minamiiwojima Island, which is estimated to be as young as 30,000 years old, it was interesting to notice that the endemic *Rubus* species on geologically younger islands showed more diverse patterns of codon usage than other insular endemics on Ulleung Island (*R. takesimensis*) and Taiwan (eight species in this study). The biased patterns toward high RSCU values of U and A, at the third codon usage in eight endemic *Rubus* plastomes in Taiwan, was similar to other angiosperms and algal lineages^40,41^.

Owing to their important function in plant metabolism, proteins and RNA molecules encoding plastid genes are subject to selective pressures^50^. While purifying selection acts to maintain protein functions, positive selection may come into play in response to environmental changes, novel ecological adaptation, or results from coevolutionary processes^50,51^. Previous studies showed that Ka/Ks values are usually less than one because synonymous nucleotide substitutions occur more frequently than nonsynonymous substitutions^52^. Interestingly, our current results from the eight endemic *Rubus* species from Taiwan suggested that ca. 40% protein-coding genes experienced positive selection pressures. All but two genes (*ndhB* and *ndhC*) of the NADH oxidoreductase genes of the eight Taiwan *Rubus* endemics showed that they were under positive selection pressure. Under strong light conditions, NADH dehydrogenase can protect plants from photoinhibition or photooxidation stress by stabilizing the NADH complex, and adjusting the photosynthetic rate and growth delay caused by drought^53,54^. In addition, all *Rubus* plastid gene encoding proteins related to transcription and post-transcriptional modification (*matK*, *rpoA*, *rpoB*, *rpoC1*, and *rpoC2*) underwent positive selection. In the same Rosaceae family, *Fragaria vesca* ssp. *vesca* showed six positively selected genes: *rpoC2, ndhD, ndhF, psbB, ycf1*, and *ycf4*^55^. We can only speculate that the positive selection pressure among eight endemic species of *Rubus* in Taiwan experienced by many genes was likely a result of their adaptation to subtropical climates in the island of Taiwan. This speculation, however, has to be investigated further.

A highly variable region or hotspot region in the whole chloroplast genome scale can help elucidate the phylogenetic relationships and complex evolutionary history of *Rubus* as a maternally inherited marker. Recently, several hot spot regions including genic and non-coding regions across the entire plastome were reported in several members of Rosaceae, such as *Malus*^56^, *Prunus*^57^, *Pyrus*^27^, and *Rosa*^30^. In our current study, we also detected four highly variable regions, including *rps16*/*trnQ* (*Pi* = 0.0518) and *petA*/*psbJ* (*Pi* = 0.0466), as two of the most variable hotspots within the eight Taiwan *Rubus* endemics, with an average nucleotide diversity (*Pi*) value of 0.01. When compared with our early study (average *Pi* value of 0.01), which included subg. *Idaeobatus* (four taxa) and subg. *Cylactis* (one taxon), the two most variable noncoding regions, *trnL*/*trnF* and *rps16*/*trnQ*, were detected with a *Pi* value of 0.05 and 0.046, respectively^35^. Yang et al.^39^ compared the four taxa of progenitor-derivative species pairs in subg. *Idaeobatus* (*R. crataegifolius*-*R. takesimensis* on Ulleung Island) and subg. *Anoplobatus* (*R. trifidus*-*R. boninensis* on the Bonin Islands), and found that the average *Pi* value (0.005) was substantially lower than that found between subg. *Idaeobatus* and subg. *Cylactis* (*Pi* = 0.01). The same study also suggested that the *trnT*/*trnL* region was the most variable region with a *Pi* value of 0.027. Thus, considering all previous studies on *Rubus* for the identification of hotspot regions throughout the complete plastomes, two intergenic regions *rsp16*/*trnQ* and *trnT*/*trnL* were found to be the most variable hotspot regions within genus *Rubus*, including members of four subgenera (*Anoplobatus, Cylactis, Idaeobatus* and *Malachobatus*. Therefore, four hotspot regions from this study, i.e., *rps16*/*trnQ*, *petA*/*psbJ*, *rpl32*/*trnL*-UAG, and *trnT*-UGU/*trnL*-UAA, can be used as effective chloroplast DNA markers for population genetic and phylogeographic studies of *Rubus* species in Taiwan.

### Phylogenetic position and relationships of Taiwanese endemic *Rubus*

Given the scarcity of available complete plastome sequences of genus *Rubus*, we were not able to meticulously assess the phylogenetic relationships among the eight endemic *Rubus* species from Taiwan and their congeneric species in Taiwan and mainland China. Nevertheless, this study provides some insights into preliminary assessments of the relationships among *Rubus* endemics in Taiwan. First of all, the clade of *Rubus* endemics in Taiwan included three species, *R. taitoensis*, *R. parviaraliifolius*, and *R. incanus*, which belong to subg. *Idaeobatus* (Fig. 7)^14^. *Rubus incanus*, which commonly occurs in open places and forest edges at medium elevation (1,800–3,000 m) throughout the central mountains in Taiwan, was treated as a synonym of *R. niveus*, which occurs widely in South Asia and Southeast Asia^3^. It was considered that *R. incanus* (narrow cymose panicles or short thyrses) and *R. niveus* (umbellate corymbs) are two distinct taxa on the basis of the inflorescence type, and our preliminary data based on the complete plastome sequences support the statement of Huang and Hu^17^ (Fig. 7). In addition, *R. incanus* has red drupelets at maturity, while *R. niveus* has red immature and black drupelets at maturity, further supporting that they are distinct taxonomic entities. *R. parviaraliifolius* occurs in low to medium altitudes (300–1,800 m) throughout the island and has 5-foliolate leaves with red fruits at maturity, and its sister species in this phylogeny, *R. taitoensis*, has simple leaves (not divided or 3-lobed) with orange to yellow fruits, occurring in medium altitudes (1,500–2,800 m) in the central mountains. It is necessary to include three species of this clade into a broader phylogenetic framework of *Rubus* in Taiwan and mainland China to precisely determine species relationships among these taxa.

The second clade includes three species in subg. *Idaeobatus*, *R. glandulosopunctatus*, *R. rubroangustifolius*, and *R. taiwanicolus*, which is more closely related to the clade of subg. *Anoplobatus* (*R. trifidus* and *R. boninensis*) and subg. *Idaeobatus* (*R. crataegifolius* and *R. takesimensis*) than other members of the same subgenus (*R*. *amabilis*, *R*. *coreanus*, *R. niveus*, *R. taitoensis*, *R. parviaraliifolius*, and *R. incanus*) (Fig. 7). In general, the clade of subg. *Idaeobatus* is not well resolved, especially subg. *Anoplobatus* deeply embedded within subg. *Idaeobatus* clade containing *R. leucanthus*, *R*. *corchorifolius*, *R. glandulosopunctatus*, *R. rubroangustifolius*, and *R. taiwanicolus*, *R. crataegifolius* and *R. takesimensis* (Fig. 7)^14^. *Rubus taiwanicolus* is a small subshrub up to 15 cm tall with 9–15 foliolate leaves, occurring in the central mountains from medium to high altitudes (1,500–3,000 m), and no previous hypothesis regarding its relationship to other congeneric species exists. In the case of *R. glandulosopunctatus*, it is considered a synonym of *R. rosifolius*, which occurs widely in Asia (East Asia, South Asia, and Southeast Asia), Africa, and Australia. As pointed out by Huang and Hu^17^, this widespread species exhibits tremendous morphological variations, requiring more investigation on this complex taxon. Given that the chloroplast and nuclear combined phylogenies (Figs. 1 and 2)^14^, *R. rosifolius* (= *R. rosaefolius*; spelling variant) is closely related to the clade containing *R. hirsutus*, *R. eustephanaus* var. *glandulinger*, and *R. tsangii* var. *linearifoliolus*. However, the target capture sequencing phylogeny suggested that *R. rosifolius* is closely related to *R. illecebrosus*, *R. trifidus*, and *R. craetaegifolius*^15^. In our current complete plastome-based phylogeny (Fig. 7), the clade containing *R. glandulosopunctatus* is closely related to the clade containing *R. trifidus* and *R. craetaegifolius*, which is consistent with the target capture sequencing phylogeny^15^. Extensive sampling regarding the phylogenetic relationships of this complex taxon still have to be further determined. *R. rubroangustifolius*, which is endemic to eastern and northern Taiwan, was treated as a synonym of *R. croceacanthus* var. *glaber*^17^. *Rubus croceacanthus* has never been included in previous phylogenetic studies, and it has been known for its tremendous morphological variations in Taiwan. In Huang and Hu^17^, *R. rubroangustifolius* was treated as a synonym of *R. cardotii,* and *R. croceacanthus* var. *glabra* was treated as a synonym of *R. cardotii*. It was also pointed out that *R. cardotii* can be easily distinguished from *R. croceacanthus* on the basis of several morphological characteristics. However, little is known about the phylogenetic position of *R. rubroangustifolius* and its relationship to *R. croceacanthus* and *R. cardotii*.

Lastly, the third clade of two endemic species of subg. *Malachobatus*, *R. laciniatostipulatus,* and *R. kawakamii* form a clade with *R. lambertianus* var. *glaber*, another species of the same subgenus. *R. fockeanus* from subg. *Cylactis* is sister to the clade of subg. *Malachobatus*. Based on three concatenated regions of chloroplast DNA among Chinese *Rubus* species, it was shown that subg. *Cylactis* is closely related to subg. *Malachobatus*, including few exceptional species from subg. *Idaeobatus* (e.g., *R. pungens* complex and *R. peltatus*) and *Dalibardastrum* (*R. amphidasys* and *R. tsangorum*). Subg. *Cylactis* has also shown to be highly polyphyletic on the basis of target capture sequencing study^15^. *Rubus foeckeanus* typically occurs in high elevation (2,000–4,000 m in glassy slopes and forests) and has 3-foliolate compound leaves. *Rubus laciniatostipulatus* occurs widely in southern China and southeastern Asia. In Taiwan, it occurs in forest edges in the northern and central parts of the island at low elevations (20–300 m)^17^. In the case of *R. kawakamii*, it is commonly distributed in forests at medium altitudes (1,000–2,500 m) throughout the central mountains. This species is very difficult to distinguish from *R. swinhoei*, which occurs at low altitudes (20–1,200 m) in northern and central parts, and is often treated as a variety of *R. swinhoei*^58^. Nevertheless, both *R. laciniatostipulatus* and *R. kawakamii* have simple leaves, shallowly 5–7 lobed or not divided, respectively, and display a close relationship^14^. Overall, phylogenetic relationships among the endemic species, including infraspecific taxa, require broader taxon sampling from China and Taiwan to gain new insights into infrageneric relationships, as well as their plastome evolution. It is also necessary to assemble plastome sequences from members of subg. *Rubus* and several other subgenera to fully understand plastome evolution and to reveal the complex evolutionary history of *Rubus* on a global scale.

## Methods

### Plant sampling, DNA isolation, and plastome sequencing/annotation

We first sampled eight out of the 15 endemic *Rubus* species in Taiwan, representing two subgenera, *Idaeobatus* (*R*. *glandulosopunctatus*, *R*. *incanus*, *R*. *rubroangustifolius*, *R*. *parviaraliifolius*, *R*. *taitoensis*, and *R*. *taiwanicolus*) and *Malachobatus* (*R*. *kawakamii* and *R*. *laciniastostipulatus*) to assemble plastome sequences. Voucher specimens were collected and deposited at SKK (Ha Eun Herbarium, Sungkyunkwan University, Korea). Fresh leaves were collected from Taiwan and dried with silica gel before DNA extraction. Total DNA was isolated by using the DNeasy Plant Mini Kit (Qiagen, Carlsbad, CA, USA) and sequenced with an Illumina HiSeq 4000 (Illumina, Inc., San Diego, CA, USA), yielding 150 bp paired-end read length, at Macrogen Corporation (Seoul, Korea). The resulting paired-end reads were assembled de novo using Velvet v. 1.2.10 with multiple k-mers^59^. The tRNAs were confirmed via tRNAscan-SE^60^. Annotation was conducted using Geneious R10^61^, and the annotated plastome sequences were submitted to GenBank: *R*. *glandulosopunctatus* (MT274118), *R*. *incanus* (MT274119), *R*. *kawakamii* (MT274120), *R*. *laciniastostipulatus* (MT274121), *R*. *parviaraliifolius* (MT274122), *R*. *rubroangustifolius* (MT274123), *R*. *taitoensis* (MT274124), and *R*. *taiwanicolus* (MT274125). The annotated GenBank format sequence file was used to draw a circular map with the OrganellarGenomeDRAW (OGDRAW) program v1.3.1.^62^.

### Comparative plastome analysis

Using mVISTA^63^ in Shuffle-LAGAN mode^64^, the eight complete plastomes from the endemic *Rubus* were compared: subg. *Idaeobatus* (*R*. *glandulosopunctatus*, *R*. *incanus*, *R*. *rubroangustifolius*, *R*. *parviaraliifolius*, *R*. *taitoensis*, and *R*. *taiwanicolus*) and subg. *Malachobatus* (*R*. *kawakamii* and *R*. *laciniastostipulatus*). The eight endemic *Rubus* plastome sequences in Taiwan were aligned with MAFFT v. 7^65^ and adjusted manually with Geneious^61^. Using DnaSP v. 6.10 software^66^, a sliding window analysis with a step size of 200 bp and a window length of 800 bp was conducted to determine the nucleotide diversity (*Pi*) of the plastome. The codon usage frequency was calculated using MEGA7^67^ with the relative synonymous codon usage (RSCU) value^68^, which is a simple measure of non-uniform usage of synonymous codons in a coding sequence. The DNA code used by bacteria, archaea, prokaryotic viruses, and chloroplast proteins was used^69^. Protein-coding genes were run using the online program predictive RNA editor for plants (PREP) suite^70^, with 22 genes as reference, based on a cut off value of 0.8 to predict the possible RNA editing sites in eight endemic *Rubus* from Taiwan. Analyses based on the complete cp genomes and concatenated sequences of 75 common protein-coding genes among the studied species were performed via MAFFT v. 7^65^ using Geneious R10^61^. Using DnaSP v. 6.10 software^66^, we calculated the Ka/Ks ratios of the eight endemic Taiwanese *Rubus* plastomes and compared them with each other.

### Phylogenetic analysis

For the phylogenetic analysis, the complete plastome sequences of 31 representative species from the family Rosaceae (11 species from *Rubus*, including *R. amabilis* (NC047211), *R*. *boninensis* (MH734123), *R*. *corchorifolius* (KY419958), *R. coreanus* (NC042715), *R*. *crataegifolius* (NC039704), *R*. *fockeanus* (KY420018), *R*. *niveus* (KY419961), *R*. *takesimensis* (NC 037991), *R. lambertianus* var. *glaber* (MH99240), *R. leucanthus* (MK105853), and *R*. *trifidus* (MK465682); six species from *Fragaria*; two species from *Rosa*; one species from *Prunus*; two species from *Pyrus*; and one species from *Prinsepia*) were aligned with MAFFT v. 7^65^ in Geneious^61^. Maximum likelihood (ML) analysis based on the best-fit model of TVM + F + R4 was conducted with IQ-TREE v. 1.4.2^71^. *Prinsepia utilis* was used as an outgroup, and a non-parametric bootstrap analysis was performed with 1000 replicates.

## Supplementary Information

Supplementary Tables.

## Data Availability

The datasets generated during and/or analyzed during the current study are available in GenBank, National Center for Biotechnology Information at (http://www.ncbi.nlm.nih.gov/genbank/), with reference numbers of MT274118–MT274125.

## References

[1] Chiang T-Y, Schaal BA (2006). Phylogeography of plants in Taiwan and the Ryukyu Archipelago. Taxon.

[2] Chiang Y-C, Huang B-H, Liao P-C (2012). Diversification, Biogeographic pattern, and Demographic history of Taiwanese *Scutellaria* species inferred from nuclear and chloroplast DNA. PLoS ONE.

[3] Hsieh TH (2002). Composition, endemism, and phytogeographical affinities of the Taiwan flora. Taiwania.

[4] Peng CI, Chung KF, Li HL, Editorial Committee of the Flora of Taiwan (1998). Compositae. Flora of Taiwan.

[5] Li HL, Lu SY, Yang YP, Tseng YH, Editorial Committee of the Flora of Taiwan (1998). Ericaceae. Flora of Taiwan.

[6] Hsieh CF, Ohashi H, Editorial Committee of the Flora of Taiwan (1993). Rubus. Flora of Taiwan.

[7] Kalkman C, Kubitzki K (2004). Rosaceae. The Families and Genera of Vascular Plants.

[8] Focke WO (1910). Species *Ruborum monographiae* generis *Rubi prodromus*. Bibliotheca Bot..

[9] Focke WO (1911). Species *Ruborum monographiae* generis *Rubi prodromus*. Bibliotheca Bot..

[10] Focke WO (1914). Species *Ruborum monographiae* generic *Rubi prodromus*. Bibliotheca Bot..

[11] Alice LA, Campbell CS (1999). Phylogeny of *Rubus* (Rosaceae) based on nuclear ribosomal DNA internal transcribed spacer region sequences. Am. J. Bot..

[12] Yang JY, Pak JH (2006). Phylogeny of Korean *Rubus* (Rosaceae) based on *ITS* (nrDNA) and *trnL/F* intergenic region (cpDNA). J. Plant Biol..

[13] Yang J, Yoon H-S, Pak J-H (2012). Phylogeny of Korean *Rubus* (Rosaceae) based on the second intron of the LEAFY gene. Can. J. Plant Sci..

[14] Wang Y, Chen Q, Chen T, Tang H, Liu L, Wang X (2016). Phylogenetic insights into Chinese *Rubus* (Rosaceae) from multiple chloroplast and nuclear DNAs. Front. Plant Sci..

[15] Carter KA (2019). Target capture sequencing unravels *Rubus* evolution. Front. Plant Sci..

[16] Wang Y (2019). Allopolyploid origin in *Rubus* (Rosaceae) inferred from nuclear granule-bound starch synthase I (*GBBI*) sequences. BMC Plant Biol..

[17] Huang J-Y, Hu J-M (2009). Revision of *Rubus* (Rosaceae) in Taiwan. Taiwania.

[18] Naruhashi N, Iwatsubo Y, Peng C-I (2002). Chromosome numbers in *Rubus* (Rosaceae) of Taiwan. Bot. Bull. Acad. Sin..

[19] Powell W (1995). Hypervariable microsatellites provide a general source of polymorphic DNA markers for the chloroplast genome. Curr. Biol..

[20] Shaw JEB (2005). The tortoise and the hare II: Relative utility of 21 noncoding chloroplast DNA sequences for phylogenetic analysis. Am. J. Bot..

[21] Sun J (2018). Phylogeny of *Maleae* (Rosaceae) based on multiple chloroplast regions: Implications to genera circumscription. Biomed. Res. Int..

[22] Kress WJ, Wurdack KJ, Zimmer EA, Weigt LA, Janzen DH (2005). Use of DNA barcodes to identify flowering plants. Proc. Natl. Acad. Sci. USA.

[23] CBOL Plant Working Group (2009). A DNA barcode for land plants. Proc. Natl. Acad. Sci. USA.

[24] Lee C, Kim S-C, Lundy K, Santos-Guerra A (2005). Chloroplast DNA phylogeny of the woody *Sonchus* alliance (Asteraceae: Sonchinae) in the Macaronesian Islands. Am. J. Bot..

[25] Jenks AA, Walker JB, Kim S-C (2011). Evolution and origins of the Mazatec hallucinogenic sage, *Salvia divinorum* (Lamiaceae): A molecular phylogenetic approach. J. Plant Res..

[26] Cho M-S (2014). Molecular and morphological data reveal hybrid origin of wild *Prunus yedoensis* (Rosaceae) from Jeju Island, Korea: Implications for the origin of the flowering cherry. Am. J. Bot..

[27] Li W (2018). Development of chloroplast genomic resources for *Pyrus hopeiensis* (Rosaceae). Conserv. Genet. Resour..

[28] Viljeon E, Odeny DA, Coetzee MPA, Berger DK, Rees DJG (2018). Application of chloroplast phylogenomics to resolve species relationships within the plant genus *Amaranthus*. J. Mol. Evol..

[29] Cho M-S, Kim JH, Kim C-S, Mejias JA, Kim S-C (2019). Sow thistle chloroplast genomes: insights into the plastome evolution and relationship of two weedy species, *Sonchus asper* and *Sonchus oleraceus* (Asteraceae). Genes.

[30] Jeon J-H, Kim S-C (2019). Comparative analysis of the complete chloroplast genome sequences of three closely related east-Asian wild roses (Rosa sect. Synstylae; Rosaceae). Genes.

[31] Xu L-S, Herrando-Moraira S, Susanna A, Galbany-Casals M, Chen Y-S (2019). Phylogeny, origin and dispersal of *Saussurea* (Asteraceae) based on chloroplast genome data. Mol. Phylogenet. Evol..

[32] Kyalo CM, Lim Z-Z, Mkala EM, Malombe I, Hu G-W, Wang Q-F (2020). The first glimpse of *Streptocarpus ionanthus* (Gesneriaceae) phylogenomics: Analysis of five subspecies’ chloroplast genomes. Plants.

[33] Zhang S-D (2017). Diversification of Rosaceae since the Late Cretaceous based on plastid phylogenomics. New Phytol..

[34] Yang JY, Pak J-H, Kim S-C (2017). The complete chloroplast genome sequence of Korean raspberry *Rubus crataegifolius* (Rosaceae). Mitochondrial DNA B.

[35] Yang JY, Pak J-H, Kim S-C (2018). The complete plastome sequence of *Rubus takesimensis* endemic to Ulleung Island, Korea: Insights into molecular evolution of anagenetically derived species in *Rubus* (Rosaceae). Gene.

[36] Chen Q (2019). The complete chloroplast genome sequence of Rubus coreanus an excellent diseases-resistant resource. Mitochondrial DNA B.

[37] Guo W, Chen Y, Deng L, Wu W (2019). The complete chloroplast genome sequence of *Rubus leucanthus* Hance (Rosaceae). Mitochondrial DNA B.

[38] Zhu Q, Tian Y, Liang W (2019). The complete chloroplast genome sequence of *Rubus eucalyptus* (Rosaceae). Mitochondrial DNA B.

[39] Yang JY, Takayama K, Pak J-H, Kim S-C (2019). Comparison of the whole-plastome sequence between the Bonin Islands endemic *Rubus boninensis* and its close relative, *Rubus trifidus* (Rosaceae), in the southern Korean Peninsula. Genes.

[40] Ravi V, Khurana JP, Tyagi AK, Khurana P (2008). An update on chloroplast genomes. Plant Syst. Evol..

[41] Morton BR (1998). Selection on the codon bias of chloroplast and cyanelle genes in different plant and algal lineages. J. Mol. Evol..

[42] Rabah SO (2017). Plastome sequencing of ten nonmodel crop species uncovers a large insertion of mitochondrial DNA in cashew. Plant Genome.

[43] Pinard D, Myburg AA, Mizrachi E (2019). The plastid and mitochondrial genomes *of Eucalyptus grandis*. BMC Genom..

[44] Kim S-H, Yang JY, Park JS, Yamada T, Maki M, Kim S-C (2019). Comparison of whole plastome sequences between thermogenic skunk cabbage *Symplocarpus renifolius* and nonthermogenic *S. nipponicus* (Orontioideae; Araceae) in East Asia. Int. J. Mol. Sci..

[45] Potter D (2007). Phylogeny and classification of Rosaceae. Plant Syst. Evol..

[46] Wolfe KH, Bogorad L, Vasil IK (1991). Protein-coding genes in chloroplast DNA: compilation of nucleotide sequences, data base entries, and rates of molecular evolution. The Photosynthetic Apparatus: Molecular Biology and Operation. Cell Culture and Somatic Cell Genetics of Plants.

[47] Kim KJ, Jansen RK (1995). *ndhF* sequence evolution and the major clades in the sunflower family. Proc. Natl. Acad. Sci. USA.

[48] Gu W, Zhou T, Ma J, Sun X, Lu Z (2004). The relationship between synonymous codon usage and protein structure in *Escherichia coli* and *Homo sapiens*. Biosystems.

[49] Nie X (2014). Comparative analysis of codon usage patterns in chloroplast genomes of the Asteraceae family. Plant Mol. Biol. Rep..

[50] Piot A, Hackel J, Christin PA, Besnard G (2018). One-third of the plastid genes evolved under positive selection in PACMAD grasses. Planta.

[51] Burri R, Salamin N, Studer RA, Roulin A, Fumagalli L (2010). Adaptive divergence of ancient gene duplicates in the avian MHC class II beta. Mol. Biol. Evol..

[52] Makałowski W, Boguski MS, Hughes AL, Yeager M (1998). Synonymous and nonsynonymous substitution distances are correlated in mouse and rat genes. J. Mol. Evol..

[53] Fan X, Zhang J, Li W, Peng L (2015). The *NdhV* subunit is required to stabilize the chloroplast NADH dehydrogenase-like complex in *Arabidopsis*. Plant J..

[54] Horvath EM (2000). Targeted inactivation of the plastid *ndhB* gene in tobacco results in an enhanced sensitivity of photosynthesis to moderate stomatal closure. Plant Physiol..

[55] Cheng H (2017). The complete chloroplast genome sequence of strawberry (*Fragaria*×*ananassa* Duch.) and comparison with related species of Rosaceae. PeerJ.

[56] Zhang X (2018). Complete chloroplast genome sequence of *Malus hupehensis*: Genome structure, comparative analysis, and phylogenetic relationships. Molecules.

[57] Katayama H, Uematsu C (2005). Structural analysis of chloroplast DNA in *Prunus* (Rosaceae): Evolution, genetic diversity and unequal mutations. Theor. Appl. Genet..

[58] Liu, F.-Y., Ou, C.-H., Chen, Y.-C., Chi, Y.-S. & Lu, K.-C. Trees of Taiwan (ed. Department of Forestry) Taichung: NCHU **1**, 120–132 (in Chinese) (2000).

[59] Zerbino DR, Birney E (2008). Velvet: Algorithms for de novo short read assembly using de Bruijn graphs. Genome Res..

[60] Lowe TM, Eddy SR (1997). tRNAscan-SE: A program for improved detection of transfer RNA genes in genomic sequence. Nucleic Acids Res..

[61] Kearse M (2012). Geneious Basic: An integrated and extendable desktop software platform for the organization and analysis of sequence data. Bioinformatics.

[62] Greiner, S., Lehwark, P. & Bock, R. Organellar genome DRAW(OGDRAW) version 1.3.1: Expanded toolkit for the graphical visualization of grnagnellar genomes. *Nucleic Acids Res.* **47**, W59–W64. 10.1093/nar/gkz238 (2019). 10.1093/nar/gkz238 PMC6602502 30949694

[63] Frazer KA, Pachter L, Poliakov A, Rubin EM, Dubchak I (2004). VISTA: Computational tools for comparative genomics. Nucleic Acids Res..

[64] Brundo M (2003). Global alignment: Finding rearrangements during alignment. Bioinformatics (19S1).

[65] Katoh K, Standley DM (2013). MAFFT multiple sequence alignment software v7: Improvements in performance and usability. Mol. Biol. Evol.

[66] Rozas J (2017). DnaSP v6: DNA sequence polymorphism analysis of large datasets. Mol. Biol. Evol..

[67] Kumar S, Stecher G, Tamura K (2016). MEGA7: Molecular evolutionary genetics analysis version 7.0 for bigger datasets. Mol. Biol. Evol..

[68] Sharp PM, Li WH (1987). The codon adaptation index—A measure of directional synonymous codon usage bias, and its potential applications. Nucleic Acids Res..

[69] Kozak M (1983). Comparison of initiation of protein synthesis in procaryotes, eucaryotes, and organelles. Microbiol. Rev..

[70] Mower JP (2009). The PREP suite: Predictive RNA editors for plant mitochondrial genes, chloroplast genes and user-defined alignments. Nucleic Acids Res..

[71] Nguyen L-T, Schmidt HA, von Haeseler A, Minh BQ (2015). IQ-TREE: A fast and effective stochastic Algorithm for estimating maximum-likelihood phylogenies. Mol. Biol. Evol..

